# Optimization of the Poly(glycerol citraconate) Synthesis Using the Box–Behnken Design

**DOI:** 10.1021/acsomega.3c00166

**Published:** 2023-05-29

**Authors:** Krzysztof Kolankowski, Julia Rżewska, Paweł Ruśkowski, Agnieszka Gadomska-Gajadhur

**Affiliations:** Faculty of Chemistry, Warsaw University of Technology, Noakowskiego 3 Street, 00-664 Warsaw, Poland

## Abstract

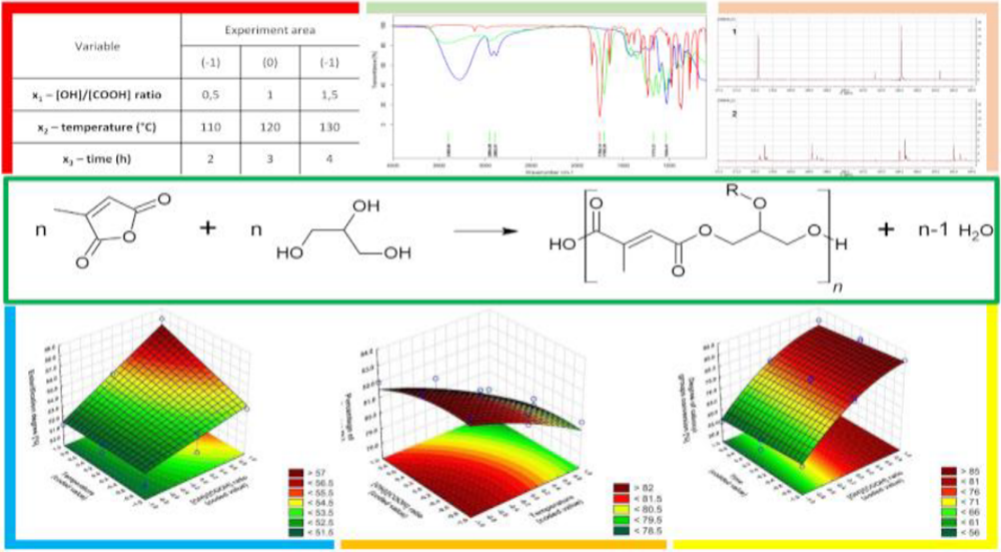

This work aimed to obtain poly(glycerol citraconate) (PGCitrn) for biomedical applications, analyze the obtained polyester by spectroscopic methods, and optimize its preparation. Polycondensation reactions of glycerol and citraconic anhydride were carried out. It was provided that the results in the reaction are oligomers of poly(glycerol citraconate). Optimization studies were carried out based on the Box–Behnken design. The input variables in this plan were the ratio of functional groups, temperature, and time and occurrence in coded form: −1, 0, or 1. Three output variables were optimized: the degree of esterification, the percentage of *Z*-mers, and the degree of carboxyl group conversion; they were determined by titration and spectroscopic methods. The optimization criterion was to maximize the values of output variables. A mathematical model and an equation describing it were determined for each output variable. The models predicted the experimental results well. An experiment was conducted under determined optimal conditions. The experimental results were very close to the calculated values. Poly(glycerol citraconate) oligomers with an esterification degree of 55.2%, a *Z*-mer content of 79.0%, and a degree of rearrangement of carboxyl groups of 88.6% were obtained. The obtained PGCitrn can serve as a component of an injectable implant. The obtained material can be used to produce nonwoven fabrics (with the addition of PLLA, for example), which can be subjected to a cytotoxicity test which can then serve as a dressing material.

## Introduction

**Figure 1:**
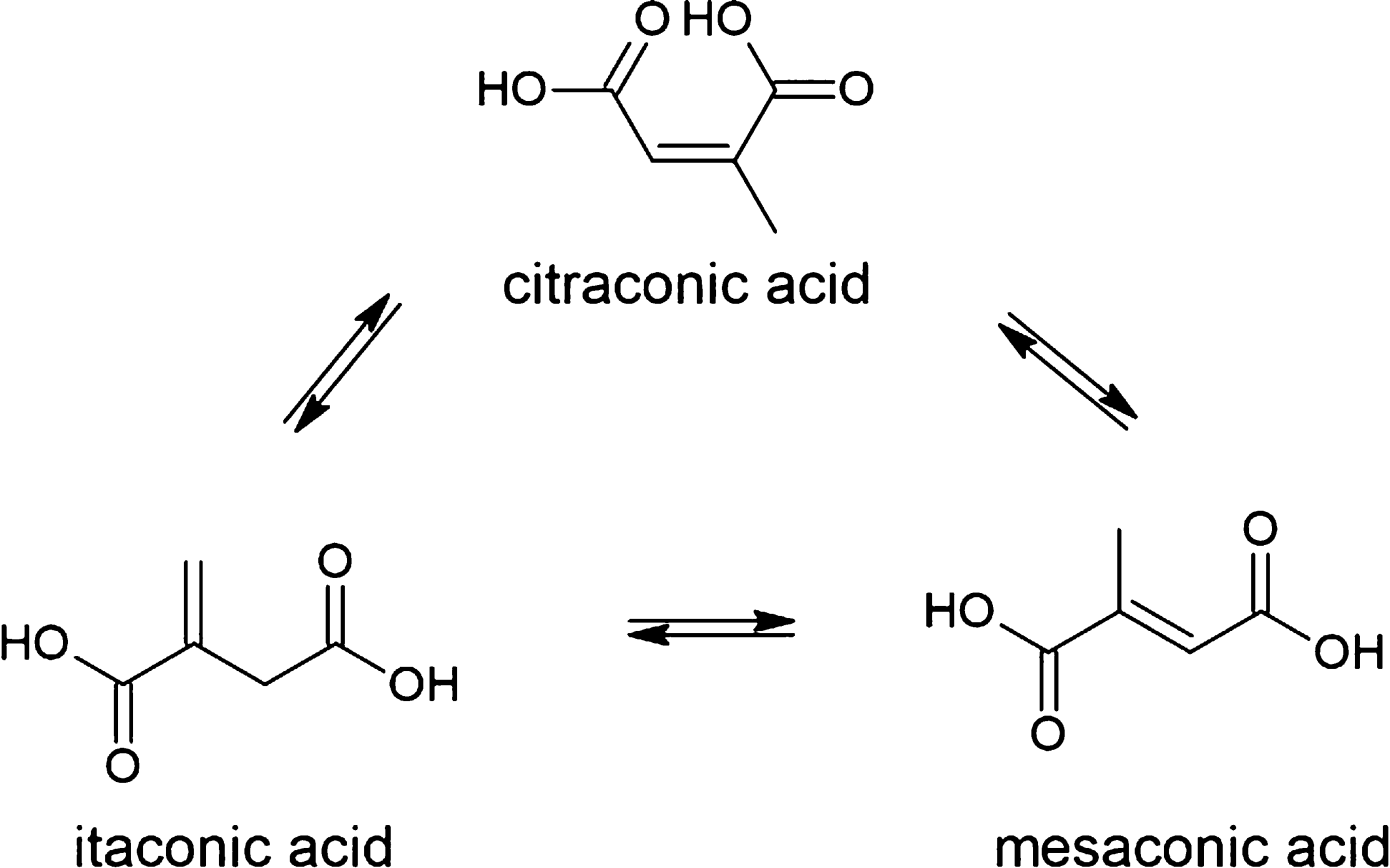
Isomerization of itaconic acid, mesaconic acid, and citraconic acid.

Glycerol-based polymers are drawing the attention of scientists worldwide, and research is being conducted on their application. Various methods of glycerol esterification have been described, leading to products ranging from linear to dendritic structures.^1−3^ Aliphatic polyesters are one of the most common biodegradable polymers used in medicine. Extensive research has been carried out in the past on their potential applications.^4−7^ All polyesters are biodegradable by hydrolysis of the ester bond.^8^ Based on the reaction conditions designated for the condensation of glycerol with other dicarboxylic acids, poly(glycerol citraconate) can be synthesized. A wide range of products with different properties, structures, and molecular weights is possible in various reaction conditions.^2,8−10^ By now, many of the glycerol polyesters are already well known. One of the most widely used is a poly(glycerol sebacate) used, for example, in heart tissue engineering,^6,11−13^ nerve tissue engineering,^6,12^ and vascular tissue engineering.^6,12^ Poly(glycerol succinate) is also a well-studied glycerol polyester.^14−16^ Materials based on it can be used in orthopedic and ophthalmic surgeries.^11^ Attempts have been made to use poly(glycerol succinate) to fabricate bonding screws for bones and as a transdermal drug delivery system.^16^ The functionalities of the received biomaterials can be further enriched using several postfunctionalization methods depending on the properties of specific cell types and tissues that are potential sites for product application.^17^ Postpolymerization modification of the products allows for obtaining functional soft materials.^18^ Poly(glycerol sebacate) nanofibers for nerve tissue reconstruction can be obtained by electrospinning.^19^ Methods of processing poly(glycerol maleate) by film fabrication,^20^ microbead manufacture,^21^ and cross-linking using amine addition^22^ are described. Optimization studies play a crucial role in the development research in technology. Among other things, they are critical when extending a process from the laboratory scale to the industrial scale.^23^ In order to optimize the manufacturing conditions of the desired product, DoE (design of experiments) is used in many fields. It plays an important role in statistical design thinking and industrial applications.^24^ First, a study design is prepared to collect the necessary data, and then the data are analyzed using a statistical method. Optimization of process parameters can lead to maximum production at minimum cost.^25^ The Box–Behnken design combines a two-stage factorial plan with an incomplete block plan. It contains coded variables appearing at three levels (−1, 0, and 1).^25^ The advantage of the Box–Behnken design is that it does not provide for experiments in which all variables simultaneously take on edge values.^23^ This avoids conducting experiments under extreme conditions. In addition, it provides an easier way to organize and analyze the results.^23,25,26^ Poly(glycerol citraconate) (Figure 3) is a polyester so far unknown in the literature. Citraconic acid is the cis isomer of 2-methyl-2-butenoic acid and often occurs together with its geometric isomer, mesaconic acid (the trans isomer).^27,28^ Citraconic acid is also an isomer of itaconic acid (Figure 1).^29^

**Figure 2:**
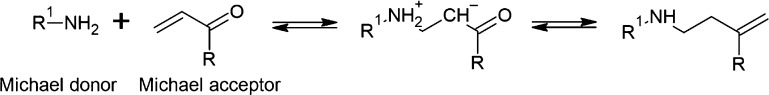
Aza-Michael addition mechanism.

Itaconic acid can polymerize by radical-initiated chain polymerization. It can also be used to produce condensation polymers such as polyesters.^29,30^ The use of amines can catalyze the isomerization of itaconic anhydride to citraconic anhydride.^31^ The presence of a carbon–carbon double bond in the structure of poly(glycerol citraconate) provides many opportunities for modification of the polyester. It can be a location for adding new side groups that can modify the final properties of the polymer.^32^ The structure of a polyester formed from citraconic anhydride includes an α,β-unsaturated ester unit, allowing the polyester to be a Michael acceptor.^30^ A widespread modification is an aza-Michael addition that occurs with amines. Aza-Michael addition is a versatile method for obtaining many valuable enhanced products.^33^ A wide range of Michael donors and acceptors are known. The process can be carried out without or with a catalyst by acids or bases.^34^ Aza-Michael addition^35^ (Figure 2) is the most direct method to generate new carbon–nitrogen bonds.^34^

**Figure 3:**
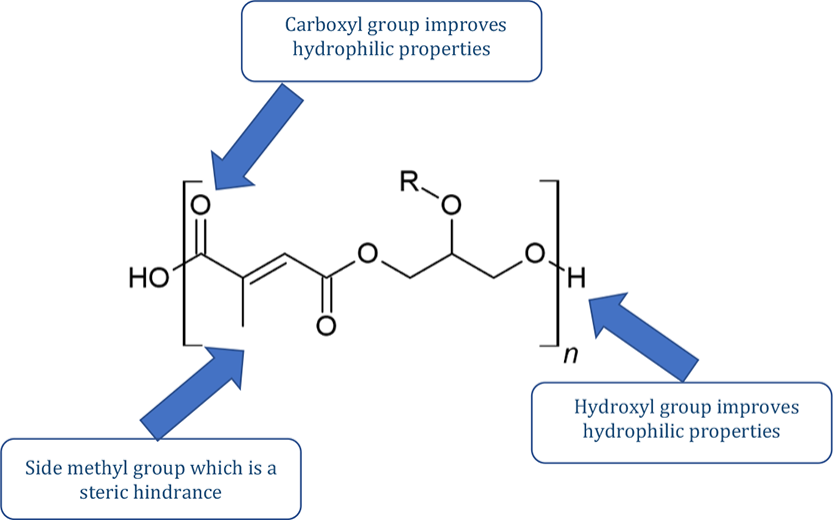
Functional groups in the poly(glycerol citraconate) structure and its characteristics.

The reaction is chemoselective, favoring cis (*Z*) groups, and trans (*E*) units are practically not involved in the reaction but do not interfere with the process.^36,37^ Due to the presence of the double bond, the poly(glycerol citraconate) particle may undergo side reactions such as isomerization or radical cross-linking.^38^ The formation of isomers increases the difficulty of polyester analysis and affects the reproducibility of the final product properties.^38^ Poly(glycerol citraconate) has a side functional group that has a small steric hindrance, which may affect the efficiency of the aza-Michael reaction. (Figure 3) The presence of functional groups such as hydroxyl and carboxyl groups can improve the hydrophilic properties of the polyester. Additionally, they affect degradation favorably and simplify modifications.^39^

**Figure 4:**
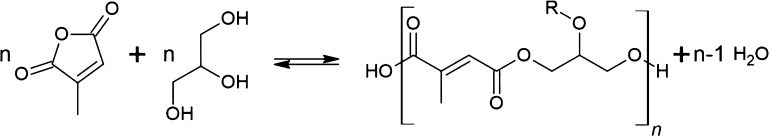
Reaction of citraconic anhydride with glycerol (R = H or PGCitrn chain).

The other advantage of PGCitn is reacting glycerol (a bulk waste from biodiesel production) and citraconic acid or anhydride (Figure 4). In addition, no toxic waste is produced during the reaction.^14−16^ Glycerol is sourced and used on a large scale, which makes it cheap. It is a substance, which allows its use in the pharmaceutical and cosmetic industries (e.g., as a plasticizer, emollient, lubricant, or moisturizer).^1,17,40^

## Materials and Methods

### Synthesis Procedure

All syntheses were carried out in a MultiMax (RB04-50) apparatus from Mettler Toledo. Appropriate amounts of glycerol (Sigma-Aldrich) and citraconic anhydride (Acros Organics) were weighed into Hastelloy reactors on a technical balance (Mettler Toledo PG4002-S) (according to Table 1). A Teflon cover was then placed on the reactor. A mechanical stirrer, temperature sensor (Pt100), and Dean-Stark apparatus were placed in the reactor cover. The setup prepared in this way was placed in a heating and cooling station operating four reactors in parallel. The device was operated via a computer.

**Table 1:** Amount of Reactants Used during the Optimization Process

[OH]/[COOH] ratio	glycerol mass [g]	citraconic anhydride mass [g]
0.5	10.75	39.25
1	17.70	32.30
1.5	22.55	27.45

This ratio of functional groups was chosen because, according to Carothers’ theory, a 1:1 ratio is the most reactive system. It was desired to test the case of a small excess of citraconic anhydride and glycerol being within the range of applicability of the theory.

### Optimization Process

Reactions were carried out according to the conditions specified in Table 2. This temperature range was chosen because it is close to the opening temperature of the anhydride ring. Conducting the synthesis for less than 2 h led to low degrees of conversion, and synthesis longer than 4 h led to a yellow-colored product - undesirable in medicine.

**Table 2:** Conditions of the Process and the Method of Coding Natural Variables

variable	experiment area		
	(−1)	(0)	(1)
*x*_1_ – [OH]/[COOH] ratio	0.5	1	1.5
*x*_2_ – temperature [°C]	110	120	130
*x*_3_ – time [h]	2	3	4

### NMR

130–160 mg of the sample was weighed on an analytical balance (RADWAG AS 220/X) and dissolved in 1 mL of deuterated DMSO. The solutions were then shaken for 24 h on a Heidolph Vortexer, after which 700 μL of the solution was transferred to a glass NMR tube.

The ^1^H NMR spectrum is an average of eight scans, and the ^13^C NMR spectrum is an average of 256 scans. 13C spectra were recorded, excluding the Nuclear Overhauser Effect (NOE). The resulting spectra were recorded using an AGILENT 400 MHz spectrometer.

### Fourier Transform Infrared (FTIR)

FTIR spectra were recorded on a BRUKER ALPHA II Platinum ATR spectrometer. The obtained spectrum is the average of 32 scans in the 400–4000 cm^–1^ range.

### Acid Number

About 1 g of the sample was weighed into a conical flask. A pipette was then used to add 25 mL of methanol. The sample was allowed to dissolve completely and titrated with NaOH with thymol blue as an indicator. The acid number was calculated according to the formula:
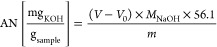
1where *V* - the volume of 1 M HCl solution used to titrate the sample. *V*_0_ - the volume of 1 M HCl used for blank titration; *M*_NaOH_ - the titer of the solution for the titration (1 M); 56.1 - the molar mass of KOH; *m* - sample weight.

Three titrations were performed for each sample, and the result is the average of them.

### Ester Number

About 0.5 g of the sample was weighed into a round-bottom flask. Then 15 mL of methanol and 20 mL of NaOH solution were added using a pipette. The sample was heated at reflux temperature under a reflux condenser for 1 h, cooled, and then titrated with HCl with phenolphthalein as an indicator. The ester number was calculated according to the formula:
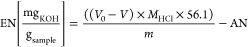
2where *V* - the volume of 1 M HCl solution used to titrate the sample. *V*_0_ - the volume of 1 M HCl used for blank titration; *M*_HCl_ - the titer of the solution for the titration (1 M); 56.1 - the molar mass of KOH; *m* - sample weight.

Three titrations were performed for each sample. The result is the average of them.

### Esterification Degree

The degree of esterification was calculated using the values of acid number and ester number
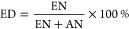
3where EN - ester number. AN - acid number. NMR calculations.

The content of *Z* formula:
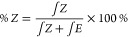
4where *Z* – the area of signals from *Z* meres of poly(glycerol citraconate) (6.28–5.82 ppm). *E* – the area of signals from *E* meres of poly(glycerol citraconate) (6.75–6.57 ppm).

The conversion of citraconic anhydride formula:
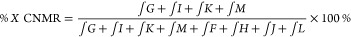
5where %*X* CNMR – degree of carboxyl groups conversion (%). *F* – the area of signals from carboxyl carbons in the molecule of citraconic anhydride. *H* – the area of signals from carboxyl carbons in the molecule of citraconic acid. *J* – the area of signals from carboxyl carbons in the molecule of citraconic anhydride. *L* – the area of signals from carboxyl carbons in the molecule of citraconic acid. *G*, *I*, *K*, *M* – areas of signals from carbons of converted carboxyl groups in a polyester molecule.

### Statistical Analysis

Statistical analysis was performed using Statistica 13 software (Statsoft Poland).

## Results and Discussion

### Spectral Analysis

**Figure 5:**
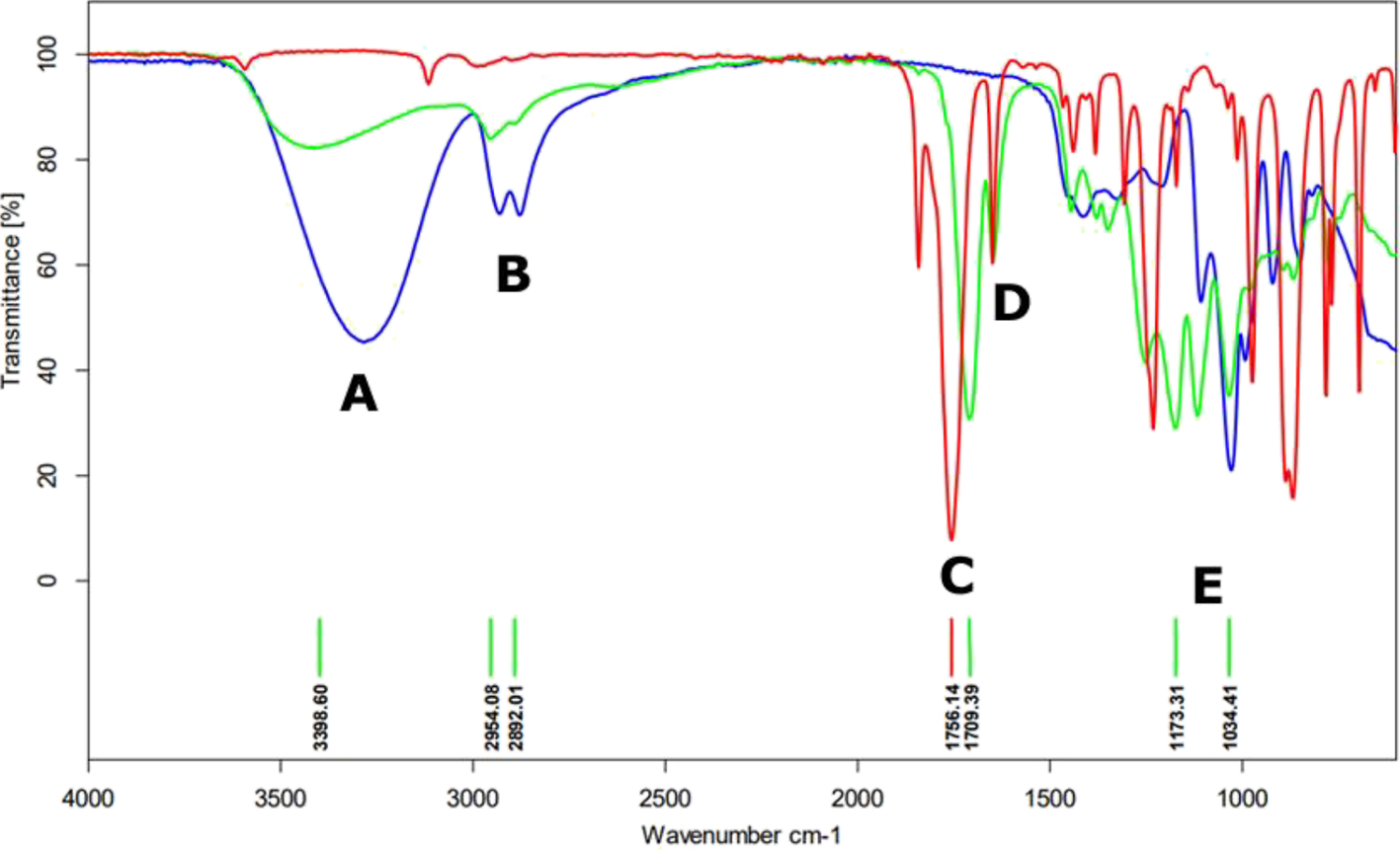
FTIR spectrum of poly(glycerol citraconate) (green), glycerol (blue), and citraconic anhydride (red).

FTIR spectrum analysis confirmed the structure of the obtained polyester (Figure 5).

A significant change in the intensity of the O–H vibration (band A) is observed, indicating its esterification. A shift toward lower wave numbers has occurred in the C=O vibration (band C)- conversion of citraconic anhydride to ester. A band labeled with the letter D indicates a double bond present in the side chain. The vibrations marked with the letter *E* are those typical of C–O bonds.

To interpret the NMR spectra of the polymer, model substances were measured. It was essential to record spectra of citraconic anhydride, citraconic acid, mesaconic acid, itaconic anhydride, itaconic acid, and glycerol (Figures S1 and S2). This allowed the development of formulas that determine, for example, the content of meres *Z* or citraconic anhydride conversion.

Ordelt saturation occurs with low efficiency. During the reaction of an unsaturated acid with alcohol, Ordelt saturation is possible as a side reaction. This results in signals visible in the proton spectrum in the 3.10–2.54 ppm range (Figure S1). Therefore, the degree of saturation of carboxyl groups is understood to be a conversion to unsaturated compounds, of which further cross-linking is possible.

**Figure 6:**
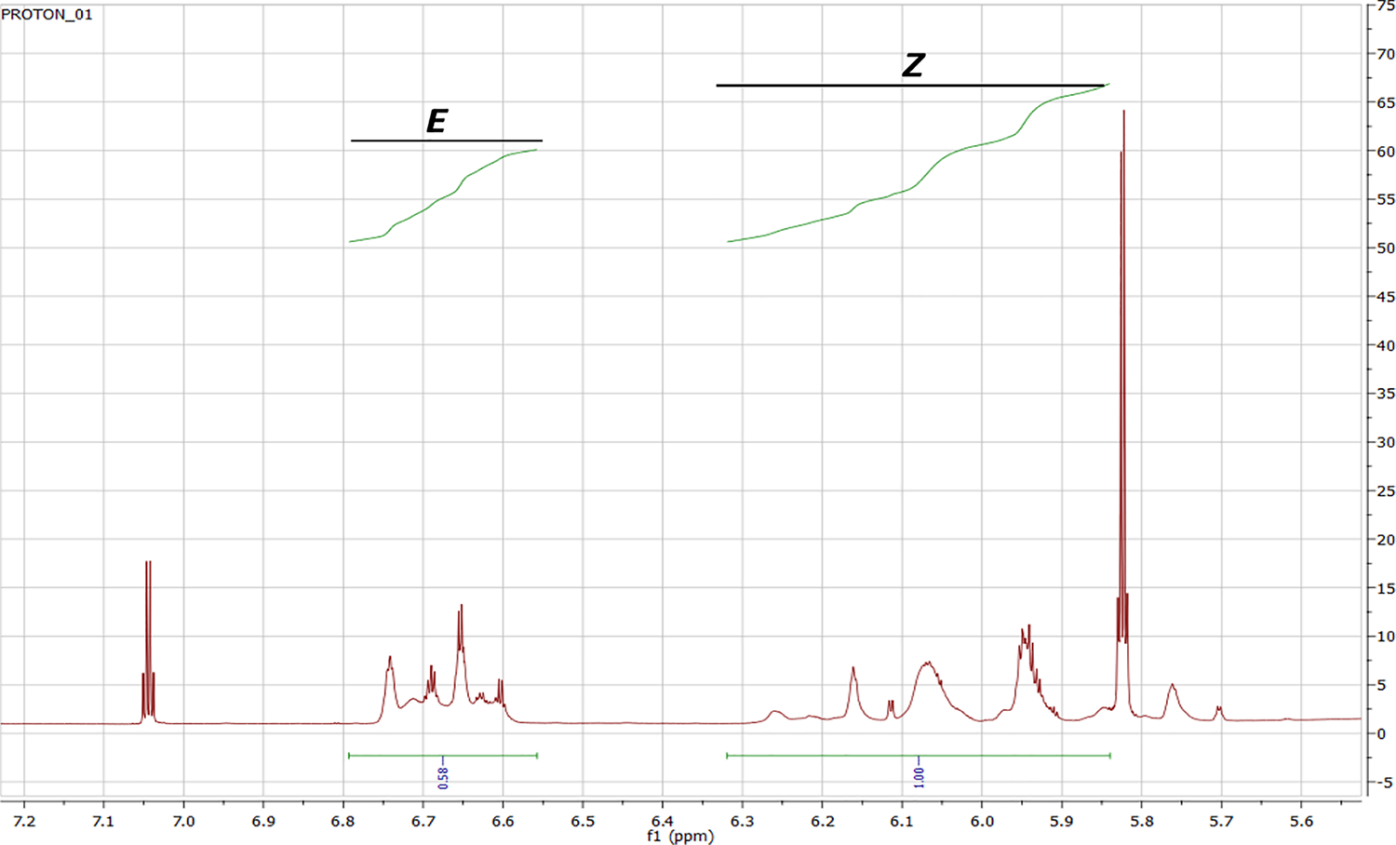
^1^H NMR spectrum of poly(glycerol citraconate) – double bond area.

The content of *Z* meres was determined based on proton spectra (Figure 6) using the formula 4 (*Z* – the area of signals from *Z* meres; *E* – the area of signals from *E* meres).

**Figure 7:**
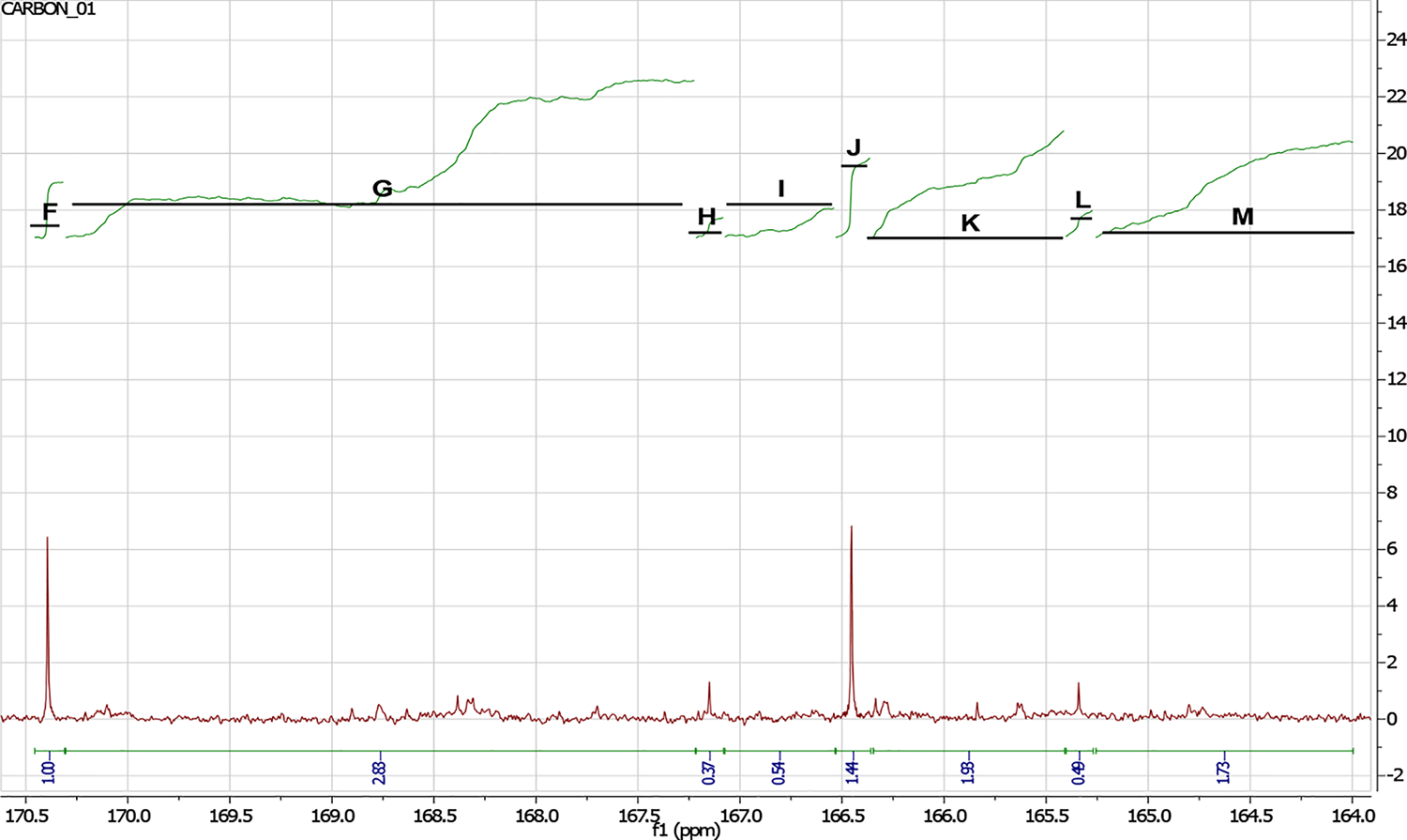
^13^C NMR spectrum of poly(glycerol citraconate) – carboxyl group area.

The conversion of citraconic anhydride was determined on the basis of carbon spectra (Figure 7) using the formula 5 (*G*, *I*, *K*, *M* – areas of signals from carbons of converted carboxyl groups in a polyester molecule; *F*, *H*, *J*, *L* – the area of signals from carboxyl carbons in the molecule of citraconic acid or citraconic anhydride).

### Statistical Analysis

Based on the NMR spectra, the values of the output variables were calculated using the values of the areas under the signals assigned to specific protons/carbons (formulas 4 and 5). The degree of esterification was determined by titration methods (1, 2, and 3 formulas). These values were entered into Statistica software, and statistical models were then created. The values of the experimental variables with the variables calculated by the models are summarized in the table below (Table 3).

**Table 3:** Experimental Values (Exp); Calculated Values (Calc); and Differential between Experimental and Calculated Values (Diff)

coded variables			esterification degree (%)			percentage of *Z* meres (%)			carboxyl group conversion degree (%)		
*x*_1_	*x*_2_	*x*_3_	Exp	Calc	Diff	Exp	Calc	Diff	Exp	Calc	Diff
–1	–1	0	50.50	50.90	–0.40	83.10	83.20	–0.10	58.00	57.40	0.60
1	–1	0	53.60	53.20	0.40	82.00	82.00	0.00	84.70	83.80	0.90
–1	1	0	51.20	50.70	0.50	80.00	79.90	0.10	53.40	54.70	–1.30
1	1	0	58.00	56.70	1.30	78.70	78.70	0.00	85.50	86.50	–1.00
–1	0	–1	51.00	50.00	1.00	82.00	81.90	0.10	58.10	57.70	0.40
1	0	–1	54.00	54.10	–0.10	80.90	80.70	0.20	86.10	86.80	–0.70
–1	0	1	51.80	51.60	0.20	81.30	81.40	–0.10	54.60	54.40	0.20
1	0	1	55.30	55.70	–0.40	80.00	80.20	–0.20	84.40	83.50	0.90
0	–1	–1	51.40	51.20	0.20	82.90	83.10	–0.20	77.20	77.50	–0.30
0	1	–1	52.00	52.90	–0.90	79.80	79.90	–0.10	78.40	77.50	0.90
0	–1	1	53.40	52.80	0.60	82.90	82.60	0.30	74.30	74.20	0.10
0	1	1	54.30	54.50	–0.20	79.40	79.40	0.00	73.20	74.20	–1.00
0	0	0	52.10	52.80	–0.70	81.80	81.80	0.00	76.10	75.90	0.20
0	0	0	51.90	52.80	–0.90	81.80	81.80	0.00	75.70	75.90	–0.20
0	0	0	53.30	52.80	0.50	81.80	81.80	0.00	76.10	75.90	0.20

Values for differences between experimental and calculated results are also included. The differences between the two are insignificant. The results of the experiments from the center of the plan (*x*_1_ = 0, *x*_2_ = 0, and *x*_3_ = 0) are similar. This shows that the exponentiation is highly reproducible and that the input variables were chosen correctly (the small influence of variables not taken into account in the model). The equations and graphs characterizing the effect of input variables on output variables are, in turn:
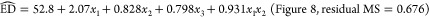

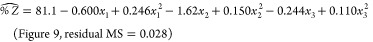

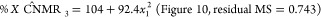


**Figure 8:**
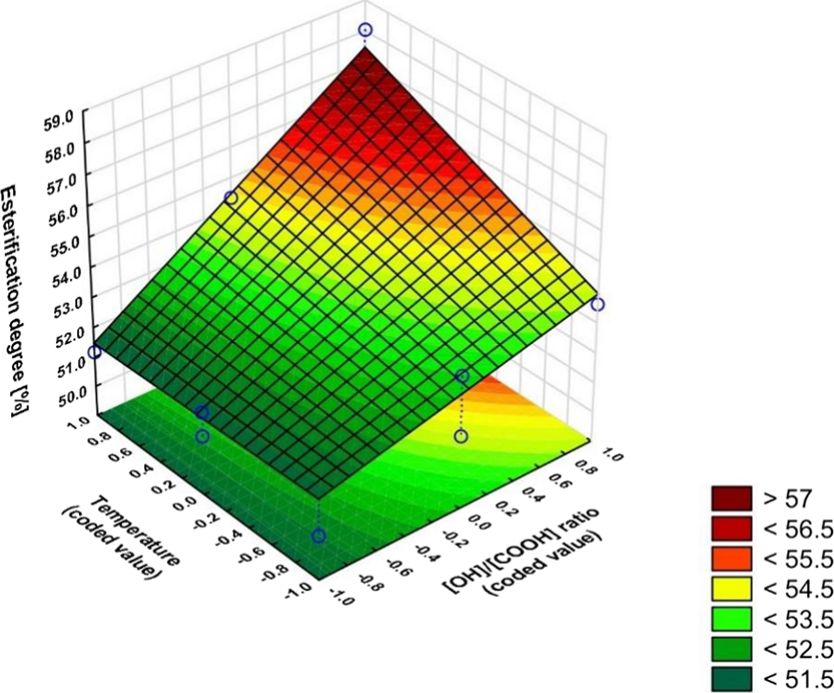
Dependence of the esterification degree on the OH/COOH ratio and temperature (*x*_3_ = 1).

**Figure 9:**
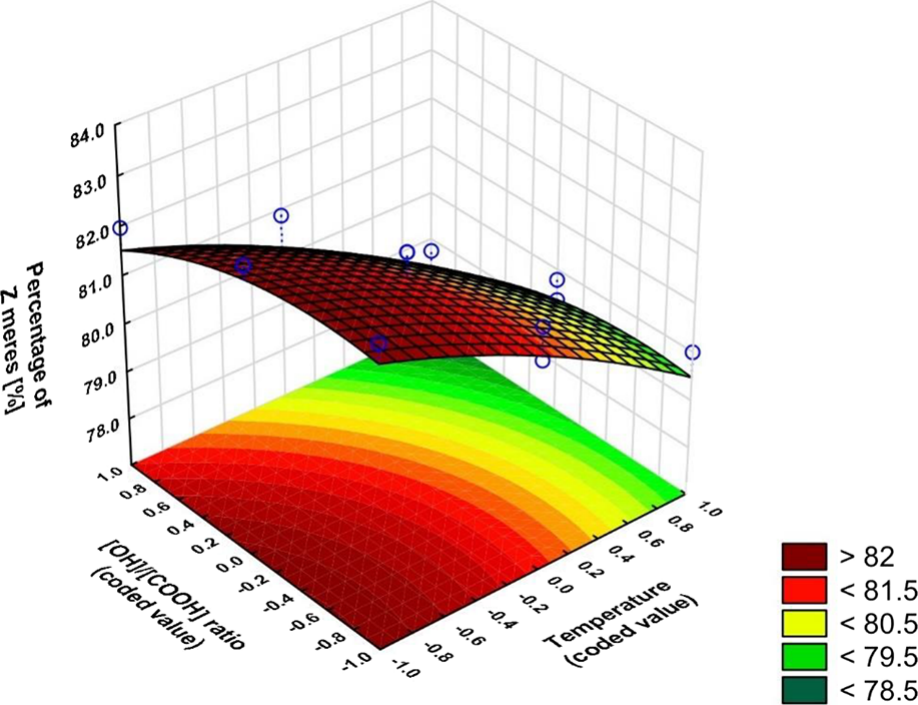
Dependence of the esterification degree on the temperature and OH/COOH ratio (*x*_3_ = 1).

**Figure 10:**
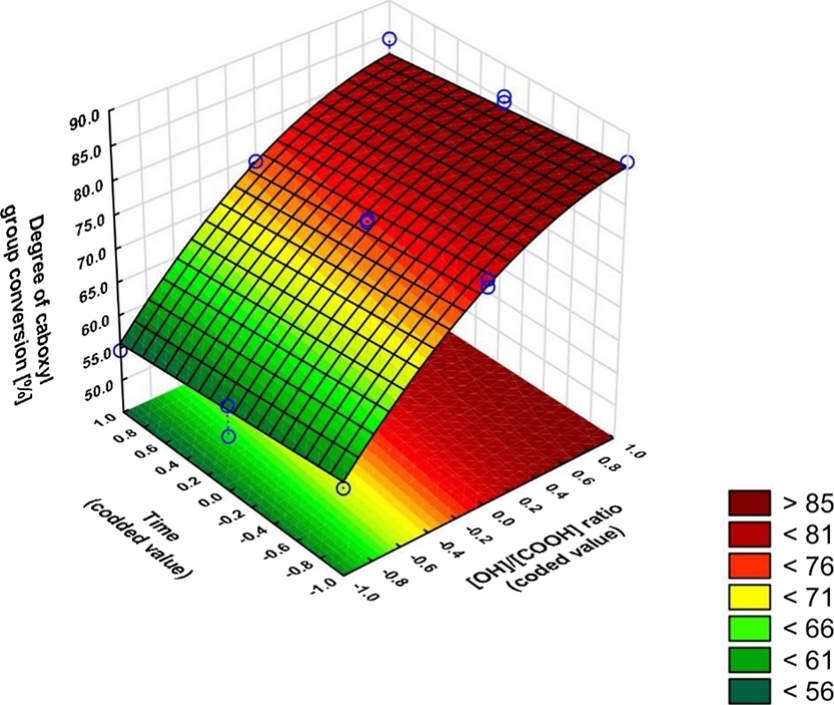
Dependence of the esterification degree on the OH/COOH ratio and time (*x*_2_ = −1).

The regression equations also included insignificant coefficients based on Pareto plots (Figure S3), as their inclusion resulted in a better model fit (Figures 8−10).

The higher the OH/COOH ratio and the higher the temperature, the higher the degree of esterification. An excess of glycerol favors the formation of linear esters (less steric hindrance around the primary hydroxyl group). An excess of glycerol results in greater anhydride conversion which results in a lower acid number which results in a higher degree of esterification. As the temperature increases, the degree of isomerization of the *Z* to *E* mers increases, which is consistent with our earlier studies.^41^Figure 10 shows how strong the effect of the ratio of OH/COOH groups is in comparison to reaction time. It is impossible to get a higher degree of anhydride conversion by running the reactions longer without changing the ratio of groups in favor of glycerol. Citraconic anhydride shows a different reactivity from itaconic anhydride. an article^42^ in which the polycondensation of itaconic anhydride and glycerol was carried out under similar conditions which showed that, in addition to the ratio of functional groups, temperature rather than time was significant.

After performing the optimization, it was determined what the impact of the input variables on the output variables was. The optimal conditions for conducting the process were determined. The profile of the utility of the responses was used. As a high utility, the highest values of the output variables were defined. The average values of the output variables were set as a medium utility, and the lowest values were set as a low utility. The Statistica program proposed conditions for the synthesis and created profiles of approximated values (Figure S4).

The optimal conditions for conducting the process in coded variables were determined and converted to natural values (Table 4).

**Table 4:** Calculated Optimal Conditions in Coded and Natural Values

	[OH/COOH] ratio	temperature	time
coded variable	1.0	–1.0	0.63
natural value	1.5	110 °C	3 h 38 min

Experimental results were compared with calculated values of output variables (Table 5).

**Table 5:** Experimental and Calculated Values

	experimental values (%)	calculated values (%)
esterification degree	55.2	55.1
percentage of *Z* meres	79.0	81.7
degree of carboxyl group conversion	88.6	84.4

Based on the above data, it can be seen that the experimental results are close to the values calculated from the models. The results obtained do not differ by more than 5% from the values calculated in the program.

We obtained a product with the targeted values of the degree of esterification, content of *Z* meres and the degree of carboxyl group conversion. The optimization was successful. It was possible to reduce the process’s time (important from a technological point of view).

## Conclusions

The optimal conditions for the synthesis of poly(glycerol citraconate) were determined. In all models, the variable with the most significant influence is the OH/COOH ratio. The experiment was carried out under these conditions, i.e., OH/COOH ratio = 1.5, temperature 110 °C, and time 3 h 38 min. In order to obtain poly(glycerol citraconate) with the highest possible degree of esterification and anhydride conversion and maximum percentage of *Z* meres, it is necessary to take under consideration the isomerization of the double bond, which is the result of high temperature and longtime of conducting the synthesis.

The experimental results of the experiment conducted under optimal conditions were very close to the values calculated by the model. Oligomers of poly(glycerol citraconate) with an esterification degree of 55.2%, a percentage of *Z* meres of 79.0%, and a degree of carboxyl group conversion of 88.6% were obtained.

The optimization process carried out was successful. The time of running the synthesis and temperature were reduced, and it was proven that the reactor load could be much cheaper. Thus, the process was made more economical, which is crucial for further upscaling and multi-ton production. The literature lacks reports on the synthesis from the substrates used during the study. Although citraconic acid is an isomer of itaconic acid, the reaction conditions using these two acids cannot be compared.

## Supporting Information Available

Method of calculating molar ratios and amounts of reactants used, ANOVA tables and *R*^2^ values, ^1^H and ^13^C NMR spectra for reactants, Pareto plots, and method of determining optimal conditions (PDF)

## Abbreviations

PGCitrn: poly(glycerol citraconate)
EN: ester number
AN: acid number
ED: esterification degree
%*Z*: the content of *Z* formula
%*X* CNMR: degree of carboxyl groups conversion
