# Supplementary material for: Optimization of the Poly(glycerol citraconate) Synthesis Using the Box–Behnken Design
Source: ACS Omega. 2023 May 29;8(23):20352–9. doi: 10.1021/acsomega.3c00166 (PMC10268020; doi:10.1021/acsomega.3c00166)
Supplement: Supplementary file 1 — ao3c00166_si_001.pdf [file ao3c00166_si_001.pdf]

SUPPLEMENTARY INFORMATION

Optimisation of the poly(glycerol citraconate) synthesis using the Box-Behnken design

*Krzysztof Kolankowski[1], Julia Rżewska[1], Paweł Ruśkowski[1], Agnieszka Gadomska-Gajadhur[1]**

[1]Faculty of Chemistry, Warsaw University of Technology, Noakowskiego 3 Street, 00-664 Warsaw, Poland;

* *Correspondence: agnieszka.gajadhur@pw.edu.pl*

Citraconic anhydride has two functional groups and glycerol has 3 functional groups. We assume that the secondary functional group is as reactive as the primary one.

Table S1. Calculations for the functional groups ratio.

[OH]/[COOH] ratio	Glycerol		Citraconic anhydride	
	[g]	[mol]	[g]	[mol]
0.5	10.75	0.1167	39.25	0.3502
1	17.70	0.1922	32.30	0.2882
1.5	22.55	0.2449	27.45	0.2449

For example for [OH]/[COOH] ratio 1:

$$\frac{3 \cdot 0.1922}{2 \cdot 0.2882} = \frac{[OH]}{[COOH]} ratio$$

Table S2. ANNOVA table for esterification degree.

input variable	SS	df	MS	F	p
x_1	34.245	1	34.245	43.680	0.001
x_1^2	1.922	1	1.922	2.451	0.178
x_2	5.481	1	5.481	6.991	0.046
x_2^2	0.974	1	0.974	1.242	0.316
x_3	5.097	1	5.097	6.501	0.051
x_3^2	0.146	1	0.146	0.186	0.684
x_1x_2	3.465	1	3.465	4.420	0.090
x_1x_3	0.063	1	0.063	0.080	0.788
x_2x_3	0.032	1	0.032	0.041	0.848
error	3.920	5	0.9349		

Table S3. ANNOVA table for percentage of Z meres.

input variable	SS	df	MS	F	p
x_1	2.881	1	2.881	88.990	0.000
x_1^2	0.900	1	0.900	27.655	0.003
x_2	20.885	1	20.885	645.127	0.000
x_2^2	0.332	1	0.332	10.257	0.024
x_3	0.477	1	0.477	14.734	0.012
x_3^2	0.178	1	0.178	5.507	0.066
x_1x_2	0.004	1	0.004	0.117	0.746
x_1x_3	0.010	1	0.010	0.301	0.607
x_2x_3	0.045	1	0.045	1.384	0.292
error	0.162	5	0.032		

Table S4. ANNOVA table for degree of carboxyl groups conversion.

input variable	SS	df	MS	F	p
x_1	1695.490	1	1695.490	2610.834	0.000
x_1^2	102.693	1	102.693	158.134	0.000
x_2	1.694	1	1.694	2.609	0.167

x_2^2	0.342	1	0.342	0.527	0.500
x_3	22.411	1	22.411	34.511	0.002
x_3^2	0.044	1	0.044	0.067	0.805
x_1x_2	7.247	1	7.247	11.159	0.021
x_1x_3	0.829	1	0.829	1.276	0.310
x_2x_3	1.253	1	1.253	1.929	0.224
error	3.247	5	0.649		

Table S5. R^2 values for output variables

Output variable	R^2 value
Esterification degree	0.9288
Percentage of Z meres	0.9937
Carboxyl groups conversion	0.9982

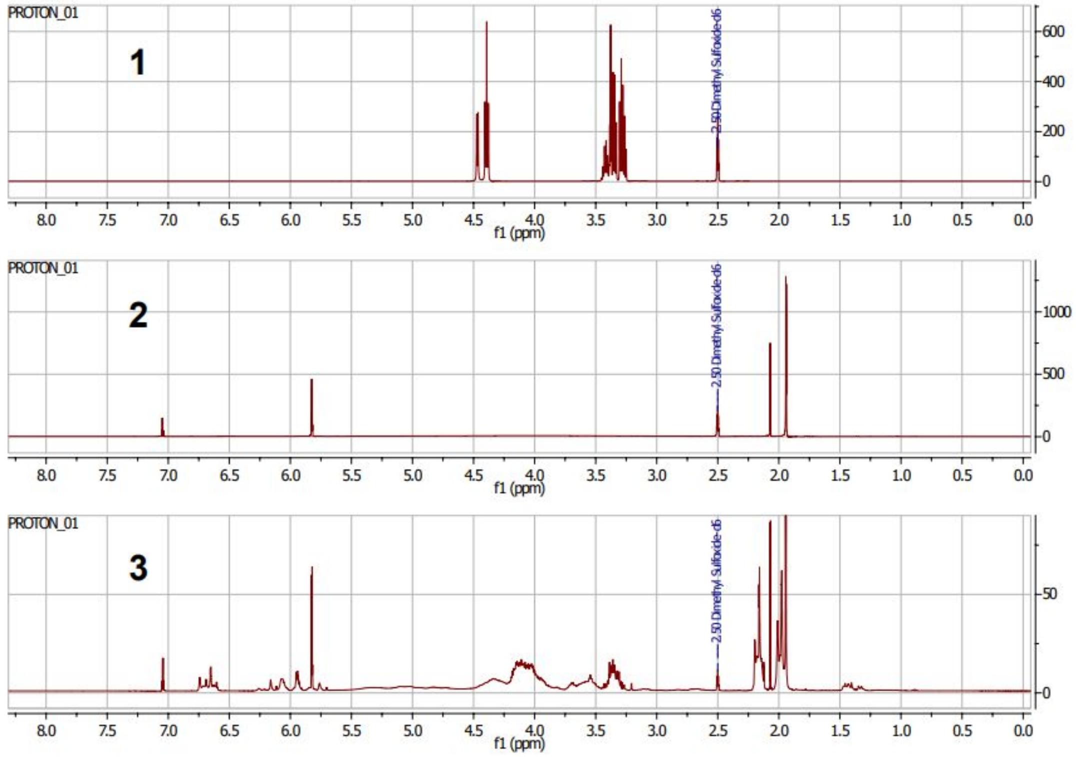

Figure S1. Comparison of ^{1}H NMR spectra; 1 - glycerol; 2 - citraconic anhydride; 3 - poly(glycerol citraconate)

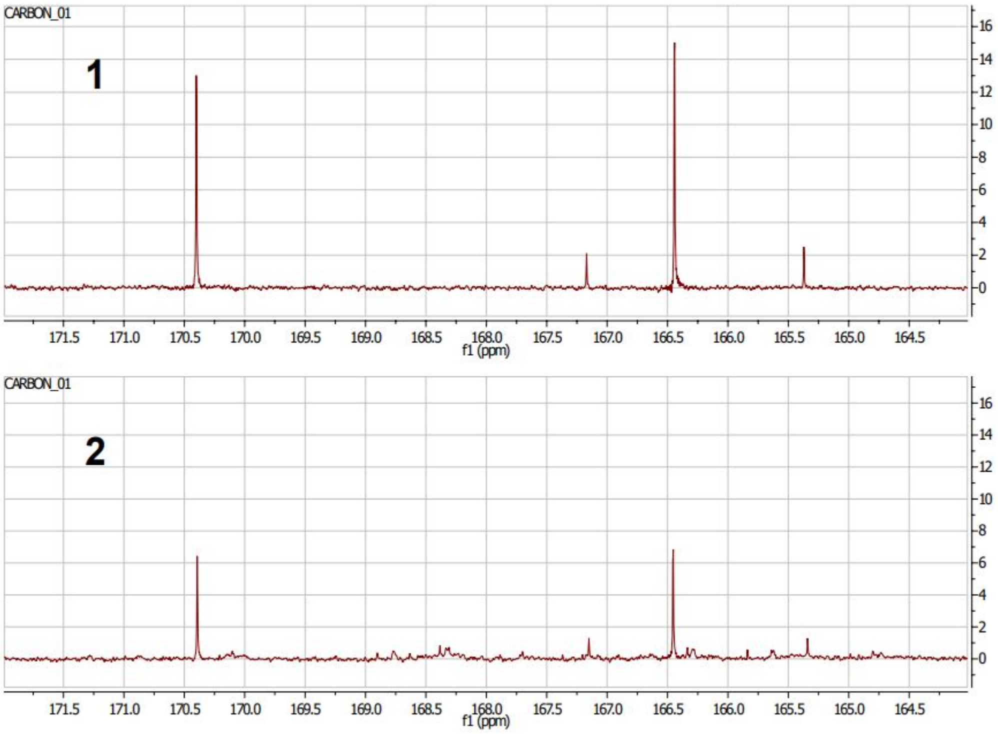

Figure S2. Comparison of ^{13}C NMR spectra; 1 - citraconic anhydride; 2 - poly(glycerol citraconate)

The confidence interval for the significance of the estimated regression coefficients was set at the 95% level hence the p-ratio is 0.05 (Fig S3). To interpret the graph, relate the absolute values of the standardized effect score to the level of the p-ratio. If they are less than or equal to the p-ratio then the variables are relevant. The regression equations also included potentially relevant coefficients as their inclusion resulted in a better model fit.

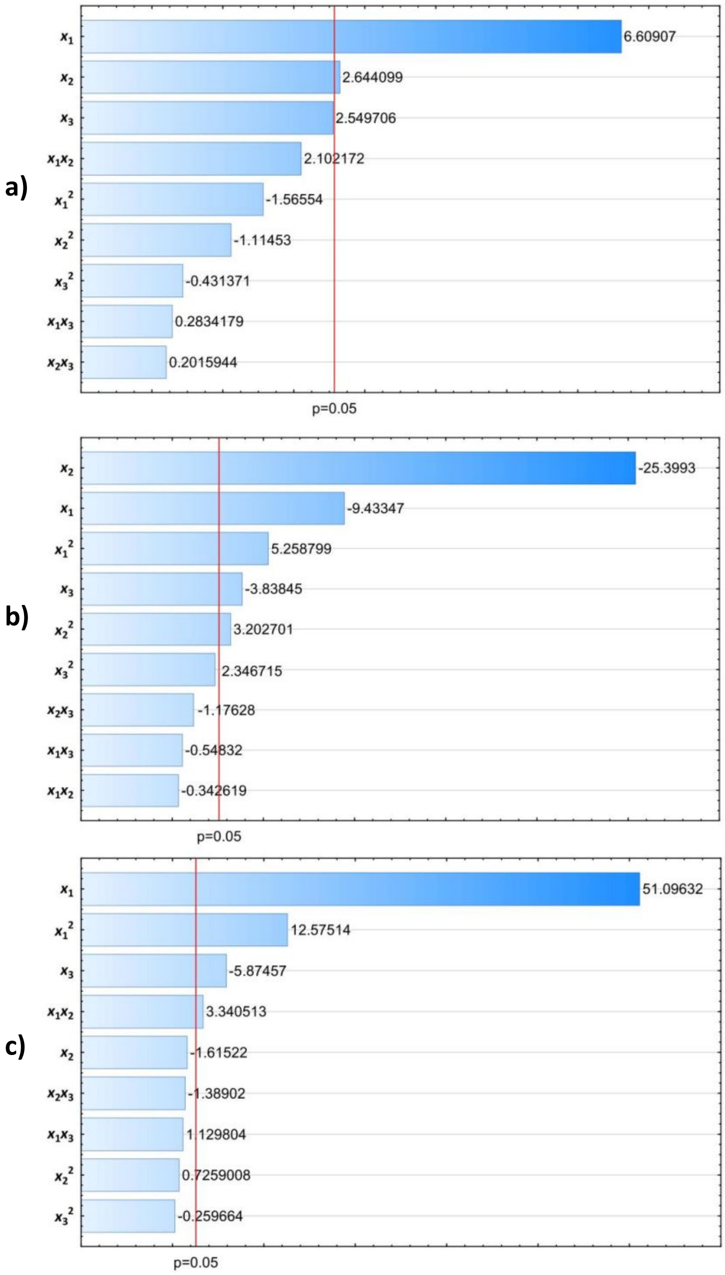

Figure S3. Pareto charts: a) ED; b) %Z; c) %X C NMR

To determine the optimal conditions for conducting the synthesis of poly(glycerol citraconate), the response utility profile was used. The highest values of the output variables were established as high utility. As values of medium utility, the medium values of the output variables, and as low utility the lowest values were set.

The program suggested conditions for conducting the synthesis and created profiles of the approximated values (Fig S4).

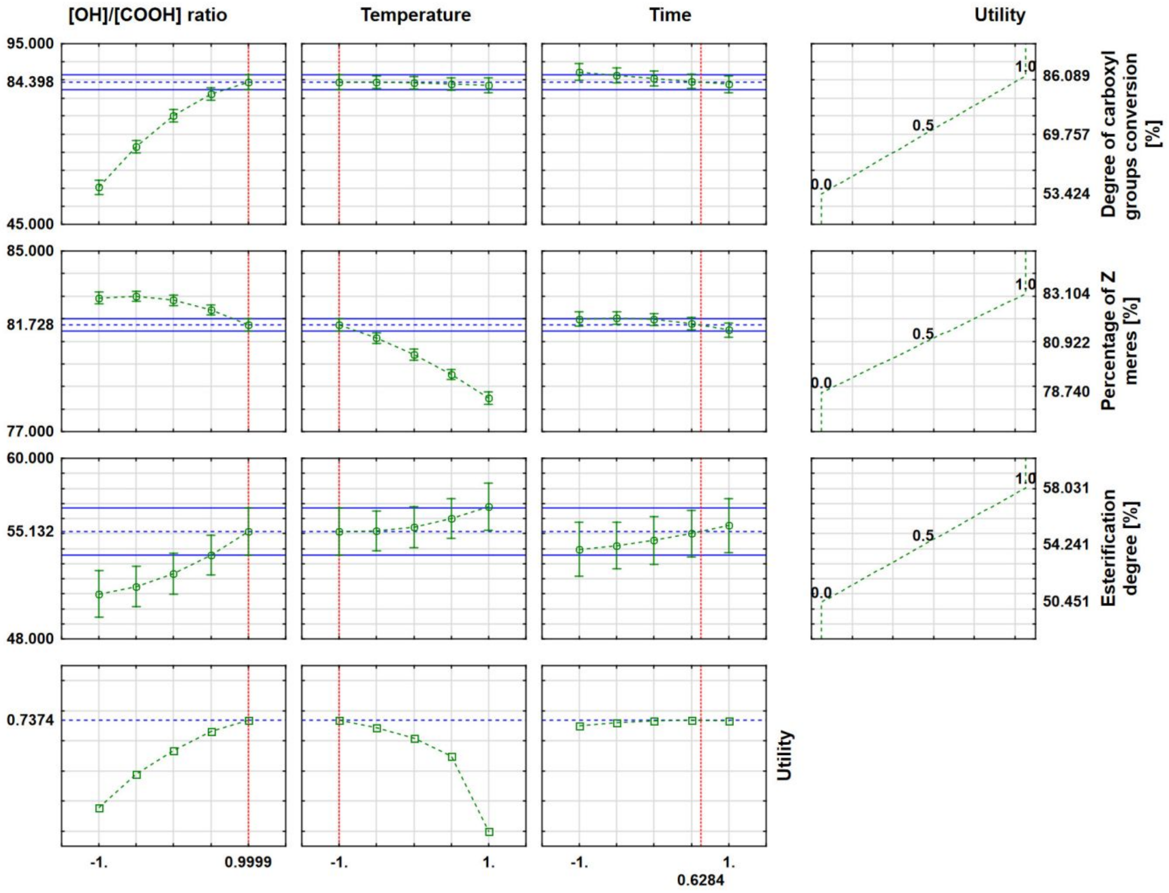

Figure S4: Optimum conditions chart.
